# Arthroscopic Removal of Loose Bodies in a Young Adult With Synovial Chondromatosis of the Shoulder: A Case Report

**DOI:** 10.7759/cureus.117575

**Published:** 2026-10-07

**Authors:** Mohamad Kassem, Hassan W Makki, Tariq A Masri-Zada, Jhamal Wallace, Vasilios Moutzouros

**Affiliations:** 1 Covenant HealthCare College of Medicine, Central Michigan University, Mount Pleasant, USA; 2 College of Osteopathic Medicine, William Carey University, Hattiesburg, USA; 3 Department of Psychiatry, Trinity Health Livonia Hospital, Livonia, USA; 4 College of Medicine, University of Illinois, Chicago, USA; 5 Department of Orthopedic Surgery, Henry Ford Health, Detroit, USA

**Keywords:** arthroscopic debridement, case report, loose bodies, synovectomy, synovial chondromatosis

## Abstract

Primary synovial chondromatosis is a rare and benign condition characterized by cartilaginous loose-body formation within a synovial joint, most commonly occurring in the knee and hip and less commonly in the shoulder and elbow. This case describes a 24-year-old Arab-American man who presented with progressive left shoulder pain that worsened over approximately one year and remained unresponsive to conservative treatment with physical therapy. Despite initial management efforts, the patient experienced increasing discomfort and functional limitation. Magnetic resonance arthrography revealed numerous intra-articular cartilaginous loose bodies. The patient underwent arthroscopic loose-body removal, synovial debridement, and synovectomy, with histopathologic examination confirming synovial chondromatosis. At the three-month follow-up, the patient demonstrated improved range of motion with pain-free abduction to approximately 160°. This case emphasizes the diagnostic challenges posed by prolonged and worsening symptoms and highlights the importance of timely specialist referral and surgical intervention in persistent cases to relieve symptoms and improve functional outcomes.

## Introduction

Primary synovial chondromatosis (SC) is a rare, benign condition affecting the synovial lining of joints and causing the formation of cartilaginous nodules that may calcify over time [1,2]. Although SC most commonly affects larger joints, such as the knee and hip, involvement of the shoulder is uncommon [2,3]. Early-stage SC can be challenging to diagnose because initial radiographs may appear normal, potentially delaying a definitive diagnosis [2,4]. The etiology of SC of the glenohumeral joint remains unknown; primary SC occurs without an identifiable underlying joint disorder [3].

Prompt recognition of SC may help prevent progressive damage to the affected joint [3]. Malignant transformation from primary SC to synovial chondrosarcoma is uncommon, with reported estimates of approximately 2.5-6.4% across retrospective institutional series; these estimates reflect different referral populations and should not be interpreted as a single established incidence [5,6]. Arthroscopy offers an alternative to the traditional open approach for loose-body removal, with potential advantages including lower morbidity, less postoperative pain, improved cosmetic results, and earlier recovery of range of motion [3,7]. Arthroscopic loose-body removal with synovectomy can provide symptom relief, although residual or recurrent synovial disease remains a concern. A systematic review of knee SC supports the use of synovectomy with loose-body removal to reduce recurrence, while shoulder-specific evidence also supports favorable clinical outcomes after arthroscopic treatment [3,8]. This case highlights the importance of considering SC in young adults with persistent shoulder pain that does not respond to conservative management, particularly when initial imaging is unremarkable.

## Case presentation

A 24-year-old right-hand-dominant man with no history of shoulder trauma, dislocation, or prior shoulder surgery presented to an orthopedic clinic with an approximately 12-month history of progressive left shoulder pain and limited abduction. He denied fever, chills, weight loss, neck pain, or neurologic symptoms and had no known history of inflammatory arthropathy or prior joint infection. The patient reported restricted mobility and pain during supine fly exercises, both weighted and unweighted.

On initial physical examination, left shoulder abduction was limited to 90° due to pain, while flexion, extension, internal rotation, and external rotation were full and symmetric to the contralateral shoulder. Shoulder strength was 5/5 throughout. These findings raised concern for rotator cuff or labral pathology. Plain radiographs obtained during the initial evaluation demonstrated preserved glenohumeral alignment and joint spaces without acute fracture, dislocation, significant degenerative change, or visible calcified intra-articular loose bodies. The patient was prescribed physical therapy consisting of isometric and active-assisted range-of-motion exercises and was instructed to apply an ice pack to the shoulder during acute pain episodes. An eight-week treatment plan was implemented to improve pain-free range of motion; however, the patient experienced no meaningful improvement.

Subsequent contrast-enhanced magnetic resonance arthrography, including the coronal oblique and axial T1-weighted images shown in Figures 1-2, revealed multiple intra-articular bodies within the subscapular recess, with the largest measuring up to 2.3 cm in greatest dimension. Numerous additional small cartilaginous loose bodies were present throughout the glenohumeral joint, particularly within the subscapular recess and axillary pouch. Erosive and cystic changes were also noted in the humeral head along the rotator interval and within the greater tuberosity near the insertion of the infraspinatus. The labrum appeared intact, making a labral tear less likely. The presence of numerous relatively uniform cartilaginous loose bodies, together with the absence of prior trauma, degenerative arthropathy, osteochondral injury, or other preexisting joint pathology, favored primary rather than secondary SC. The erosive and cystic changes were interpreted as chronic pressure-related osseous remodeling from repeated contact with intra-articular loose bodies rather than an underlying degenerative process. Rheumatoid factor testing was negative. No additional targeted laboratory evaluation for inflammatory disease or mycobacterial infection was performed, and no mycobacterial cultures were obtained.

**Figure 1:**
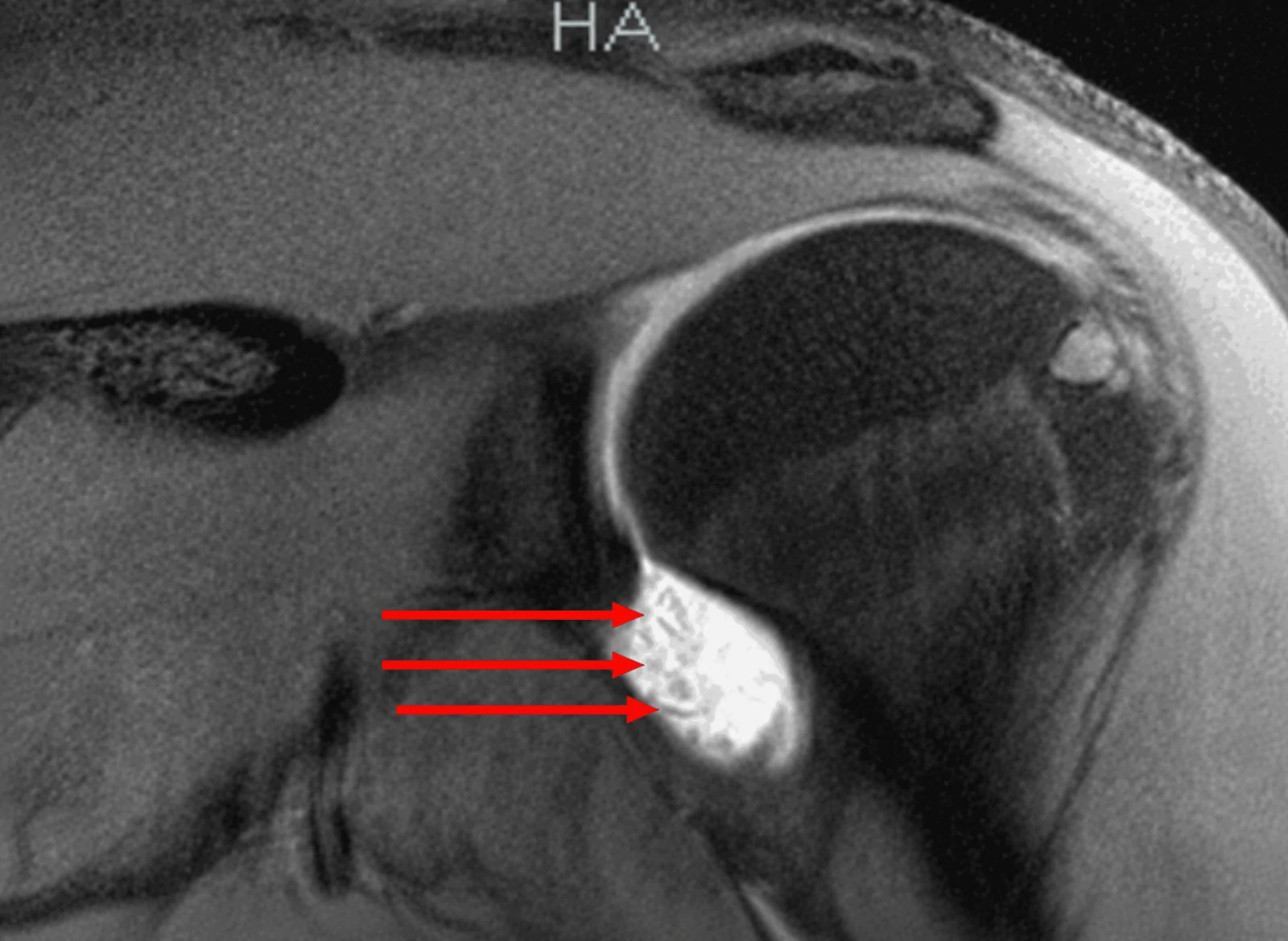
T1-weighted coronal oblique magnetic resonance arthrogram of the left shoulder with contrast Red arrows indicate intra-articular loose bodies in the axillary pouch.

**Figure 2:**
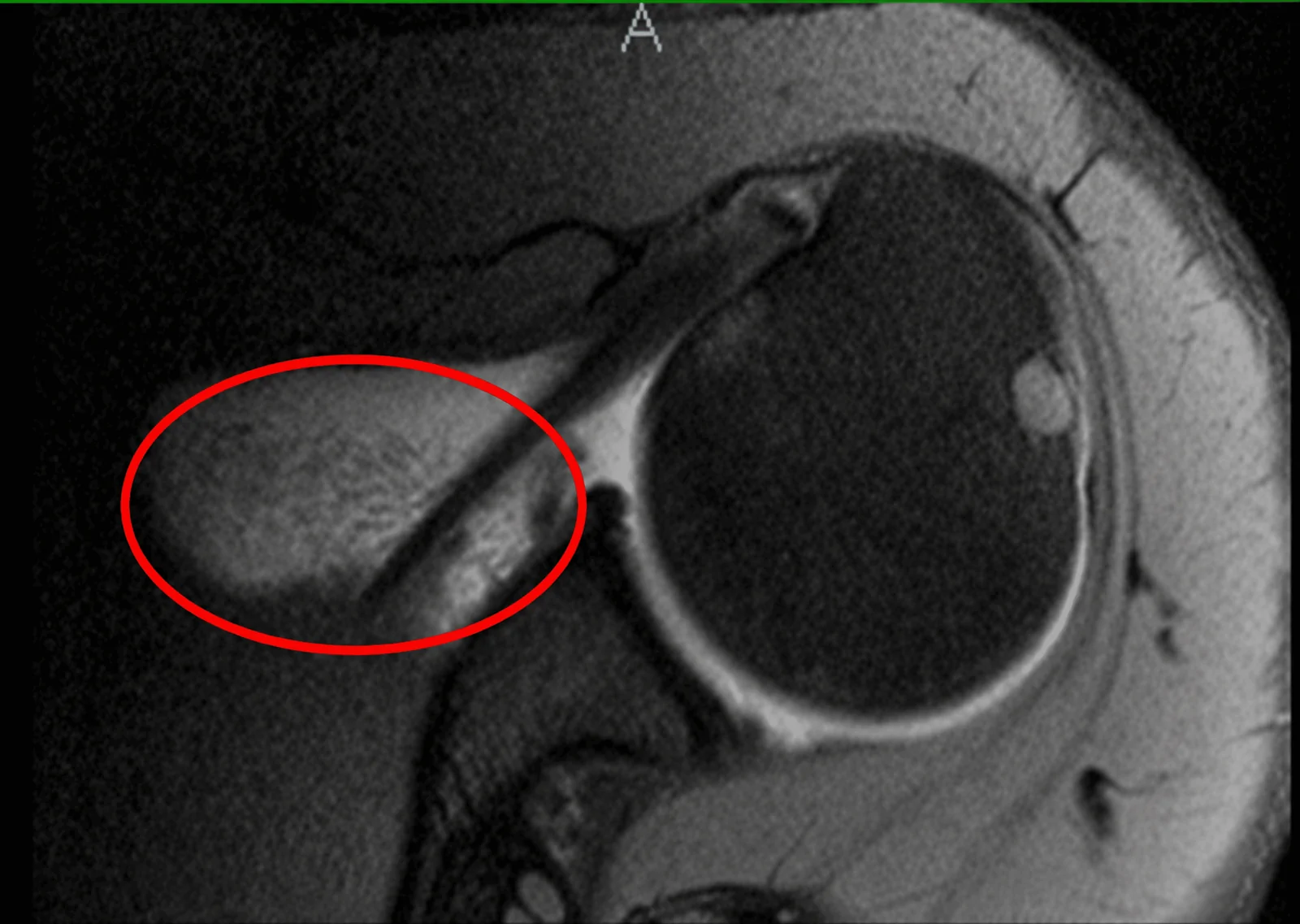
T1-weighted axial magnetic resonance arthrogram of the left shoulder with contrast Low-signal-intensity foci within the red oval represent intra-articular loose bodies consistent with synovial chondromatosis.

Because the patient's symptoms persisted despite completion of an eight-week course of physical therapy, arthroscopic loose-body removal with synovectomy was planned. Arthroscopic debridement was performed through a posterolateral portal and an anterior portal positioned just lateral to the coracoid process. Numerous loose bodies were removed from the glenohumeral joint, primarily from the axillary pouch and subscapular recess, as shown in Figure 3. Gross examination of the excised material described multiple tan fragments with the appearance of cartilaginous tissue. The approximate number and size range of the excised bodies were not documented. Complete removal of loose bodies was believed to have been achieved. Taken together, the imaging findings, intraoperative identification of numerous loose bodies, absence of underlying joint pathology, and histopathologic confirmation of their cartilaginous composition supported classification as primary SC.

**Figure 3:**
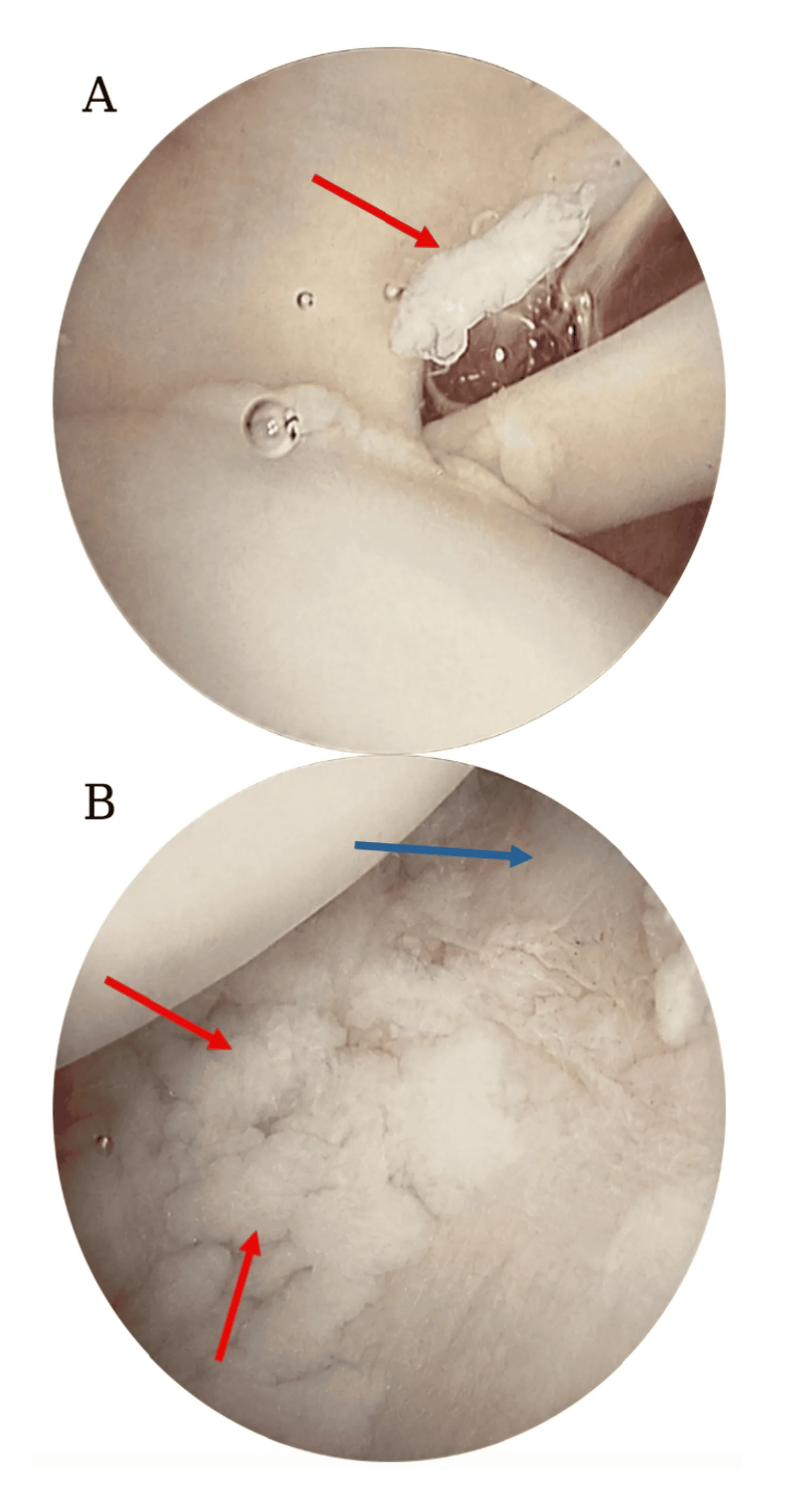
Left shoulder arthroscopy (A) Arthroscopic image demonstrating the removal of a pale cartilaginous loose body from the glenohumeral joint using a grasper. The red arrow indicates the loose body. (B) Arthroscopic view of the axillary pouch demonstrating clustered, lobulated cartilaginous loose bodies indicated by red arrows. The blue arrow indicates the villous synovial tissue.

Following the removal of loose bodies, a complete arthroscopic synovectomy of the glenohumeral joint was performed, including the axillary recess and posterior synovium, where scar tissue and inflamed synovium were identified and removed. Additional debridement of the rotator interval was performed. Final arthroscopic inspection demonstrated no residual loose bodies. The patient was placed in a simple sling postoperatively and began physical therapy to restore shoulder motion and function.

Final histopathologic examination demonstrated cartilaginous loose bodies consistent with SC. Further microscopic details were not provided in the available pathology report. No intraoperative or postoperative complications occurred. At the three-month follow-up, the patient reported improved shoulder function and demonstrated pain-free abduction to approximately 160°, compared with 90° preoperatively. Additional range-of-motion measurements, pain severity scores, return-to-activity status, and standardized functional outcome measures were not recorded, and follow-up beyond three months was unavailable. Continued physical therapy was recommended.

## Discussion

Primary SC is a rare, benign condition affecting the synovial lining of joints and leading to the formation of cartilaginous nodules that may or may not calcify over time [1,2]. Although SC typically involves larger joints, such as the knee and hip, shoulder involvement is uncommon [2,3]. SC is usually monoarticular and can occur at any age, but it most often presents during the third to fifth decades of life [9]. The age of this 24-year-old patient is therefore within the typical range.

Early diagnosis of SC may be difficult because patients frequently present with nonspecific symptoms, including joint pain, swelling, stiffness, and restricted range of motion [4]. Radiographs may fail to identify SC during the early stages because the cartilaginous loose bodies have not yet calcified [2]. Magnetic resonance imaging is more sensitive for detecting noncalcified loose bodies and provides a more comprehensive assessment of the synovium, joint capsule, and surrounding structures [4]. In this case, initial radiographs were unremarkable, whereas subsequent contrast-enhanced magnetic resonance arthrography with coronal oblique and axial T1-weighted images demonstrated multiple intra-articular cartilaginous bodies.

Differentiating SC from other joint disorders is important for selecting appropriate management. Alternative clinical considerations included a labral tear, rheumatoid arthritis, mycobacterial infection, chondrosarcoma, and pigmented villonodular synovitis (PVNS). A labral tear was initially suspected based on shoulder pain and restricted motion, but was considered less likely after magnetic resonance arthrography demonstrated an intact labrum.

Rheumatoid arthritis was considered because of the erosive changes involving the humeral head, but was considered less likely given the isolated shoulder involvement, absence of other reported clinical features of systemic inflammatory arthritis, and negative rheumatoid factor testing. These findings did not definitively exclude inflammatory arthritis, and no further targeted laboratory evaluation was performed. Mycobacterial infection was also a clinical consideration; however, no targeted infectious laboratory testing or mycobacterial cultures were obtained. The absence of systemic symptoms alone could not exclude an indolent mycobacterial process. Histopathologic examination of the excised loose bodies demonstrated cartilaginous tissue consistent with SC, supporting the final diagnosis.

Imaging features concerning for malignant transformation include medullary bone invasion and infiltrative extension into adjacent soft tissues, particularly when accompanied by rapid clinical progression or early recurrence. Although increasing lesion size may also raise suspicion, neither size nor the morphology or uniformity of loose bodies alone reliably distinguishes SC from secondary chondrosarcoma. Cortical erosion and extracapsular extension may also occur in benign disease [10]. PVNS is characterized by synovial hypertrophy and often demonstrates a hemosiderin-related blooming artifact on magnetic resonance imaging, which can help differentiate it from SC [11]. In the present case, the imaging findings, absence of an aggressive mass, arthroscopic findings, and histopathologic confirmation of cartilaginous loose bodies collectively supported the diagnosis of primary SC.

Surgical removal of loose bodies, through either an open or arthroscopic approach with synovectomy, remains the definitive treatment for symptomatic SC. Treatment aims to relieve symptoms, restore joint function, and reduce the risk of progressive cartilage damage and secondary osteoarthritis caused by persistent loose bodies [12,13]. The arthroscopic approach allows systematic evaluation and treatment of multiple shoulder compartments where loose bodies may occur, including the glenohumeral joint and the long head of the biceps tendon sheath, allowing less postoperative pain and a shorter rehabilitation period than with an open approach [3,12]. In this case, arthroscopy was selected to provide access to multiple regions of the glenohumeral joint while minimizing surgical morbidity.

Recurrence rates after surgical treatment of SC vary by involved joint, surgical technique, extent of synovectomy, completeness of loose-body removal, and follow-up duration [8,9]. In a shoulder-specific series, Liu et al. identified recurrence in three of 22 shoulders (13.6%) that underwent postoperative imaging after arthroscopic treatment [3]. The broader literature also reports recurrence rates of approximately 15-25%, particularly after incomplete excision [10]. A systematic review of knee SC also emphasizes the relevance of synovectomy when evaluating recurrence after arthroscopic loose-body removal [8]. These estimates should be interpreted within their respective populations and follow-up periods. Long-term surveillance is important because residual loose bodies or persistent abnormal synovium may lead to recurrent disease [8].

Malignant transformation of primary SC is uncommon, with reported estimates ranging from approximately 2.5% to 6.4% across retrospective institutional series [5,6]. McCarthy et al. reviewed 155 cases and identified four with aggressive behavior and chondrosarcoma-like histology, reported as approximately 2.5%: three involved the hip and one the elbow [5]. Evans et al. identified five malignant transformations among 78 patients (6.4%) treated at a supraregional orthopedic oncology referral center: four involved the hip and one the knee [6]. The higher estimate in the Evans et al. series may reflect differences in cohort size, referral population, anatomic case mix, disease severity, and case ascertainment. Neither series establishes a shoulder-specific transformation risk. Although the mechanism of SC recurrence remains incompletely understood, reactivation of pluripotent mesenchymal cells and renewed cartilaginous differentiation in response to bone morphogenetic protein signaling have been proposed [1].

This report is limited by its single-patient design and relatively short three-month follow-up, which precludes meaningful assessment of long-term recurrence. Validated preoperative and postoperative patient-reported outcome measures were unavailable, and additional objective follow-up data were not recorded. Nevertheless, this case demonstrates the diagnostic value of magnetic resonance imaging when radiographs are unremarkable and illustrates early functional improvement following arthroscopic loose-body removal and synovectomy.

## Conclusions

This case demonstrates that SC of the shoulder, although uncommon, should be included in the differential diagnosis of young adults with persistent shoulder pain that does not respond to conservative treatment, particularly when initial radiographs are unremarkable. Magnetic resonance imaging can help identify noncalcified intra-articular loose bodies and help differentiate SC from other causes of shoulder pain, although definitive diagnosis may require clinical correlation and histopathologic confirmation.

Arthroscopic removal of loose bodies combined with synovectomy provided symptomatic relief and early functional improvement in this patient. Continued clinical surveillance is warranted because recurrence may occur, and the three-month follow-up reported in this case is insufficient to establish long-term disease control. Prompt diagnosis and appropriate surgical management may help limit progressive joint deterioration, as suggested by prior literature, although this cannot be established from the short follow-up of the present case.
